# Clinical Perspective on 2019 Novel Coronavirus Pneumonia: A Systematic Review of Published Case Reports

**DOI:** 10.7759/cureus.8488

**Published:** 2020-06-07

**Authors:** Rikinkumar S Patel, Neev Patel, Mizba Baksh, Annam Zaidi, Jaiminkumar Patel

**Affiliations:** 1 Psychiatry, Griffin Memorial Hospital, Norman, USA; 2 Medicine, Byramjee Jeejeebhoy Medical College, Ahmedabad, IND; 3 Internal Medicine, Dr. Nandamuri Taraka Rama Rao University of Health Sciences, Vijayawada, IND; 4 Medicine, Dow University of Health Sciences, Karachi, PAK; 5 Internal Medicine, Albert Einstein College of Medicine, Bronx, USA

**Keywords:** multi-viral pneumonia, interstitial pneumonia, covid-2019, covid-19 pneumonia, novel corona virus, corona pandemic, ards

## Abstract

The ongoing pandemic of 2019 novel coronavirus (2019-nCoV), which originated from Wuhan, China, has led to 68,279 deaths due to 2019-nCoV pneumonia as of May 5, 2020. We conducted a systematic review and included 16 case reports to summarize the transmission and pathology of 2019-nCoV, and clinical presentation, laboratory and imaging findings, and treatment in 2019-nCoV pneumonia. The disease is mild in most people; in some, it may progress to severe pneumonia with acute respiratory distress syndrome (ARDS). Patients with mild illness usually recover at home, with supportive care and isolation in accordance with guidelines. Patients who have moderate to severe pneumonia are usually monitored in the hospital. Although there is no definitive treatment for 2019-nCoV pneumonia so far, some antiviral drugs have shown promising results. The use of lopinavir/ritonavir and remdesivir was associated with significant clinical improvement in severe pneumonia. Nonetheless, we need more randomized clinical trials (RCTs) and treatment guidelines for developing effective management of the 2019-nCoV and improve patient outcomes by reducing mortality in high-risk patients. We also need ﻿more clinical trials and management guidelines for the effective management of 2019-nCoV pneumonia.

## Introduction and background

The ongoing outbreak of the 2019 novel coronavirus (2019-nCoV) has posed significant threats to international health and the economy [1]. In late December 2019, a cluster of patients were admitted to hospitals with an initial diagnosis of pneumonia of an unknown etiology. These patients were epidemiologically linked to the seafood and wet animal wholesale market in Wuhan, Hubei Province, China [2].

Analysis of the viral genome has revealed that the new coronavirus (CoV) is phylogenetically close to severe acute respiratory syndrome coronavirus (SARS-CoV), the causative agent of a viral outbreak in 2002. Thus, the new CoV has been named SARS-CoV type 2 by the International Committee on Taxonomy of Viruses (ICTV) and other virologists [3]. On February 11, 2020, the World Health Organization (WHO) Director-General announced that the disease caused by this new CoV was called as coronavirus disease 2019 (COVID-19).

This new virus seems to be very contagious and has quickly spread globally. On March 11, 2020, as the number of 2019-nCoV cases outside China has increased 13 times and the number of countries involved has tripled with more than 118,000 cases in 114 countries and over 4,000 deaths, WHO declared the 2019-nCoV a pandemic [4]. As of May 5, 2020, there is around 1,171,510 total number of both confirmed and probable 2019-nCoV cases in the United States (US), the highest in the world, and the total number of deaths is approximately 68,279, the majority caused by pneumonia [5].

Person-to-person transmission of SARS-CoV-2 occurs primarily through close contact with an infected person, mainly via respiratory droplets and after touching contaminated objects. Additional routes of transmission are currently under investigation, including fecal viral shedding [6]. The most common presenting symptoms are fever, cough, sore throat, breathlessness, fatigue, and malaise. The disease is mild in most people; in some (usually the elderly and those with comorbidities), it may progress to pneumonia, acute respiratory distress syndrome (ARDS), and multiorgan dysfunction [7].

In this systematic review based on findings of previously published case reports on pneumonia in 2019-nCoV, we have broadly summarized the transmission and pathology of 2019-nCoV, and demographics, clinical presentation, laboratory, and chest imaging findings, along with treatment and outcomes in 2019-nCoV pneumonia patients.

## Review

Study search strategy and selection

**Figure 1:**
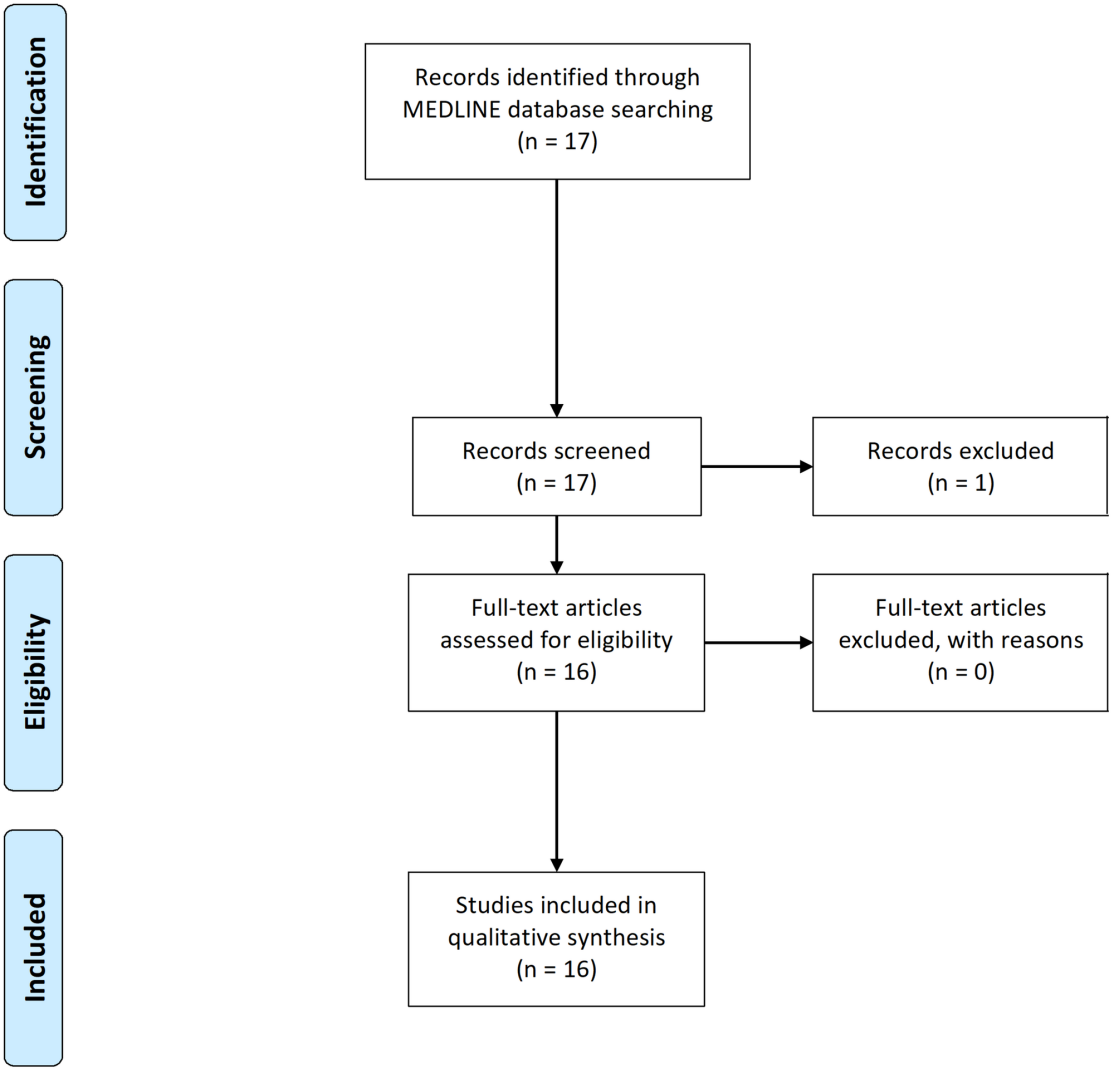
Results of systematic review

The MEDLINE database from the National Library of Medicine (NLM) was used to identify case reports published in English from December 1, 2019 to April 6, 2020. The search strings in title/abstract were ‘COVID-19’ or ‘coronavirus’ or ‘2019-nCoV’ and ‘pneumonia’ that yielded 17 articles. All searches and screening were done independently by two authors (R Patel, J Patel) using the preferred reporting items for systematic reviews and meta-analyses statement (PRISMA) recommendations. The titles and abstracts were screened, based on the purpose of our review, and resulted in the exclusion of one article. A total of 16 case report articles met the criteria for our systematic review and were included as shown in Figure 1.

Pathology and transmission

Cases of COVID-2019 pneumonia are caused by a CoV called SARS-CoV-2, which is a novel type of beta-CoV. The 2019-nCoV is the seventh member of the CoV family that includes Middle East respiratory syndrome CoV (MERS-CoV) and SARS-CoV [8]. Most early patients had a history of exposure to the south China seafood market in Wuhan. The source of infection is proposed to be the Chinese rufous horseshoe bat*, *yet the exact source of the virus is still unknown [8-10].

One study of two patients highlights the use of a low-input metagenomic next-generation sequencing (mNGS) approach on ribonucleic acid (RNA) extracted from bronchoalveolar lavage fluid. It rapidly identifies the 2019-nCoV, which was the sole pathogen in the sample, with a very high abundance level (1.5% and 0.62% of total RNA sequenced). Based on the results of genome mapping, data revealed extremely active CoV replication in the lungs of patients [10].

Transmission of 2019-nCoV is mainly via droplets, but it can also be through contact with one another (within six feet distance). In an analysis of 75,465 2019-nCoV cases in China, the airborne transmission was not reported [11]. The incubation period of 2019-nCoV is generally no longer than 14 days, with a median time of four to five days from exposure to onset of symptoms, and the patient is infective during the incubation period [8].

Elderly patients or those with underlying diseases are more seriously affected during the 2019-nCoV pandemic, whereas low infection rate and mild symptoms have been noted in the pediatric population, which could potentially be explained by greater innate immunity early in life and less exposure compared to adults [12]. Elderly 2019-nCoV infected patients are more likely to have bacterial infections, which can be a contributing factor to a higher case fatality rate in the elderly [13]. Asymptomatic individuals may transmit 2019-nCoV to other people, but it remains to be determined how common are such transmissions. Transmission dynamics and the full spectrum of clinical illness are yet to be fully understood [14].

Pathophysiology

2019-nCoV is comprised of a single-stranded RNA structure that belongs to the Coronavirinae subfamily. Sequence analysis of SARS-CoV-2 has shown a structure typical to that of other CoV and its genome has been likened to a previously identified CoV strain that caused the SARS outbreak in 2003 [15]. 2019-nCoV shared 98.7% nucleotide identity to bat CoV strain BtCoV/4991 and 87.9% nucleotide identity to bat-SL-CoVZC45 and bat-SL-CoVZXC21 strains, indicating that it was quite divergent from the currently known human CoV, including SARS-CoV [10].

SARS-CoV-2, a single-stranded RNA-enveloped virus, targets cells through the viral structural spike (S) protein that binds to the angiotensin-converting enzyme 2 (ACE2) receptor. Following receptor binding, the virus particle uses host cell receptors and endosomes to enter cells. A host type 2 transmembrane serine protease (TMPRSS2) facilitates cell entry via the S protein [16]. ACE2 is predominantly expressed by epithelial cells of the lung, intestine, kidney, heart, and blood vessels. A recent study by Liu et al. showed that serum angiotensin II levels in patients with 2019-nCoV pneumonia was significantly higher compared with healthy individuals and were linearly associated with viral load and lung injury [13]. Based on this, it can be postulated that SARS-CoV-2 binding to ACE2 may attenuate residual ACE2 activity, skewing the ACE/ACE2 balance to a state of heightened angiotensin II activity leading to pulmonary vasoconstriction and inflammatory and oxidative organ damage, which increases the risk for acute lung injury [17].

Microscopic findings included diffuse alveolar damage with exudates [18]. The inflammation was predominantly lymphocytic, and multinucleated giant cells were seen alongside large atypical pneumocytes, although no definitive viral inclusions were noted [19].

Demographics

This study included published 16 case reports (N = 22), patients were from China, Korea, Taiwan, Canada, and the US. All patients with 2019-nCoV were studied for the development of pneumonia. Patients were males (N = 13) and females (N = 9) with an age range from 10 to 75 years.

Clinical presentation

The most common clinical presentation of 2019-nCoV infection includes fever, fatigue, and dry cough; some patients present with nasal congestion, runny nose, myalgia, and chills. Headache, confusion, chest tightness, pleuritic chest discomfort, rhinorrhea, sore throat, hemoptysis, vomiting, abdominal discomfort, constipation, and diarrhea have been reported but are less common [9,10,20-22]. Some patients with 2019-nCoV have experienced gastrointestinal symptoms, such as diarrhea and nausea, prior to developing fever and lower respiratory tract signs and symptoms [14]. In one case report, the patient complained of psychiatric symptoms, such as depression, insomnia, and suicidal thoughts after isolation, due to stress regarding people's reactions from the media reports about the 2019-nCoV patients [23].

Most patients came to visit the outpatient clinic and/or emergency department (ED) after three to five days of onset of symptoms. In some patients’ symptoms gradually worsened with exertional dyspnea, high-grade fever, and type I respiratory failure [10,20,24]. In severe cases, dyspnea usually occurs one week after the onset of symptoms, and some patients can rapidly progress in 8-12 days to ARDS. The disease course of 2019-nCoV pneumonia was similar in most cases with the exception of two patients with severe 2019-nCoV pneumonia who developed complications such as ARDS, septic shock, and multiple organ dysfunction syndromes (MODS) requiring non-invasive ventilation [22,25]. There was one case from Korea of a 10-year-old girl, who was in close contact with her uncle and mother who were confirmed to have 2019-nCoV. She presented with mild pneumonia on chest CT and recovered completely by only placing her in a negative isolation unit [12].

In patients with a history of fever, cough, or contact with the epidemic area combined with significant chest CT findings, timely detection of the 2019-nCoV is required to ensure early diagnosis, isolation, and treatment [8]. During the early stages of infection, one cannot predict progression from upper respiratory infection to severe 2019-nCoV pneumonia. Considering this, it is safer to do a 2019-nCoV screening test for all symptomatic patients with epidemiological risk than to wait until full-blown pneumonia develops [9].

Imaging results and lab findings

Chest CT scan is a highly sensitive diagnostic tool to detect pneumonia because the chest x-ray (CXR) could be normal in those patients with mild or no symptoms. The most common finding on chest CT was ground-glass opacities, involving bilateral lungs with peripheral distribution noted in most patients; interlobular septal thickening and consolidation are common associated findings. As 2019-nCoV pneumonia progresses, an increase in size and density of the ground-glass opacities or crazy-paving patterns were reported [24]. One case demonstrated that the ground-glass opacities and consolidations will decrease in size as a patient recovers, though the fibrotic changes may remain as a sequela after recovery [20]. Subpleural opacities were also observable with lesions limited to lower lobe lung [8,9,12,26-28]. With a high-resolution chest CT, it will be easier to find ground-glass opacities in the early stage [26].

A few cases reported abnormalities on chest CT when CXR was normal, which led to an early diagnosis [9,23,26]. However, the chest CT imaging pattern is non-specific and overlaps with other infections, making the diagnostic value of chest CT imaging for 2019-nCoV low and dependent upon interpretations from individual radiologists [29]. Chest CT should not be used as a first-line test for screening or diagnosis and should be reserved for hospitalized, symptomatic patients with specific clinical indications. Given the variability in chest imaging findings, chest radiograph or chest CT alone is not recommended for the diagnosis of 2019-nCoV [22]. Confirmation with the viral test is required, even if radiologic findings are suggestive of 2019-nCoV on CXR or chest CT [30].

In throat-swab specimens from the upper respiratory tract that were obtained from all patients, 2019-nCoV was confirmed by real-time reverse transcriptase-polymerase chain reaction (RT-PCR) [10]. Detection of SARS-CoV-2 RNA in the blood may be a marker of severe illness [31]. SARS-CoV-2 RNA has also been detected in stool samples. Test for respiratory panel (including respiratory syncytial virus, parainfluenza virus, rhinovirus, adenovirus, metapneumovirus, Mycoplasma pneumoniae, Chlamydia pneumoniae, Legionella pneumophila, and CoV strains like HKU1, NL63, 229E, OC43) and influenza A and B were all negative [8,10,14,21,26].

Lymphopenia is the most common lab finding in 2019-nCoV patients. In one case, a detailed laboratory investigation revealed lymphopenia and elevated aspartate aminotransferase (AST), alanine aminotransferase (ALT), c-reactive protein (CRP), and lactate dehydrogenase (LDH), which may be associated with greater illness severity [20,22]. Laboratory findings in one of the patients showed that there were elevated blood levels of CRP, erythrocyte sedimentation rate (ESR), and D-dimer level [27]. Some other findings on laboratory results were elevated creatine kinase (CK), elevated alkaline phosphatase (ALP), increased level of hematocrit, increased neutrophil count, decreased lymphocyte count, increased lymphokine, and thrombocytopenia [14,21,24,26].

Treatment and outcomes

The milder spectrum of pneumonia caused by 2019-nCoV suggests that the identification of individuals who could be managed by being quarantined at home, rather than in a hospital, might be an important strategy for containing the 2019-nCoV pandemic [21]. Patients who have mild illness usually recover at home, with supportive care and isolation in accordance with guidelines. Patients who have moderate or severe diseases are usually monitored in the hospital [32]. Treatment modalities for all the patients included isolation in a negative pressure room and supportive care. Such isolation rooms can control airflow to prevent viruses from escaping into the rest of the hospital. Preferred empirical antibiotics were ceftriaxone, amoxicillin/clavulanate, and tabaxin, meropenem and linezolid, and antiviral medications included oseltamivir and ganciclovir, and interferon inhalation [20,26,27,33,34].

One of the cases that was tested positive for 2019-nCoV was isolated in negative pressure room and treated with antiviral medication: lopinavir (LPV): 200 mg/capsule, ritonavir (RTV) 50 mg/capsule twice a day (BID). Interestingly, the next day CoV load monitored by quantitative real-time RT-PCR decreased significantly to no detectable titers [23]. One study found that four 2019-nCoV positive patients were administered antiviral treatment, including LPV/RTV, arbidol, and Shufeng Jiedu Capsule (SFJDC, a traditional Chinese medicine) and supplemental oxygen. After the treatment, three patients gained significant improvement in pneumonia symptoms, and routine blood analysis revealed that leukocytes and lymphocytes were increased indicating restoration of the immune system, and they were confirmed 2019-nCoV negative and discharged. Only one of the four patients developed severe pneumonia and received immunoglobulin therapy with intubated ventilator-assisted breathing therapy due to refractory low blood oxygen pressure, and this patient also showed signs of clinical improvement [22].

One of the cases received supportive treatment initially and later on received vancomycin and cefepime. This patient developed severe pneumonia with high 2019-nCoV viral load, and so received antiviral therapy with intravenous remdesivir (a novel nucleotide analog prodrug in development) on day 7, and the next day, patient’s clinical condition significantly improved [14]. When LPV/RTV was used in high-risk groups of 2019-nCoV pneumonia, it showed reduced viral loads and improvement in clinical symptoms during the treatment [23]. LPV/RTV regimen has shown substantial clinical benefit in China and is currently recommended along with SFJDC by National Health Commission of the People’s Republic of China [35]. However, observational studies done during an epidemic often do not have concurrent controls, have a significant risk of bias, and use surrogate outcomes like viral clearance rather than patient-important outcomes [36]. Most of the patients in our systematic review demonstrated a good prognosis with no complications. However, two patients had developed ARDS and MODS. All the patients, at the end of hospitalization, were cured and discharged in stable condition.

Currently, the Centers for Disease Control and Prevention (CDC) panel recommends against the use of combination of LPV/RTV or other human immunodeficiency virus (HIV) protease inhibitors for the treatment of 2019-nCoV pneumonia, except in the context of a clinical trial [37]. On the contrary, remdesivir has been recently recognized as a promising antiviral drug against a wide array of RNA viruses (including SARS/MERS-CoV5) infection in cultured cells, mice, and non-human primate models [38]. In vitro studies showed that remdesivir can inhibit SARS-CoV and MERS-CoV replication, and in an in vitro test utilizing epithelial cell cultures of a primary human airway, remdesivir was effective against bat-CoV, pre-pandemic bat-CoV, and circulating contemporary human-CoV in primary human lung cells. Remdesivir improved pulmonary function, reduced lung viral loads, and ameliorated severe lung pathology [39]. In contrast, prophylactic LPV/RTV-interferon-β (IFN-β) reduced viral loads and did not impact other disease parameters, and therapeutic LPV/RTV-IFN-β improved pulmonary function but did not reduce virus replication or severe lung pathology. Overall, these results indicated that remdesivir showed more potential than LPV/RTV-IFN-β for treating MERS-CoV infections [40]. Also, a systematic review on acute respiratory distress patients, who were positive for 2019-nCoV, was managed with LPV/RTV with or without oseltamivir, out of which the majority of them showed significant improvement [41]. 

Patients with advanced 2019-nCoV pneumonia who received remdesivir recovered faster than similar patients who received a placebo, according to a preliminary data analysis from the first randomized controlled trial (RCT) involving 1,063 patients in the US [42]. Recently, the US food and drug administration (FDA) issued an emergency use authorization (EUA) allowing for remdesivir to be distributed in the US and administered by healthcare providers, as appropriate, to treat suspected or confirmed 2019-nCoV positive adults and children with severe disease [43].

An overview of the included case reports is shown in Table 1.

**Table 1:** Summary of included case reports of 2019 novel coronavirus pneumonia COVID-19: coronavirus disease 2019; b/l: bilateral; CXR: chest x-ray; WBC: white blood cell; BID: twice daily; IV: intravenous; CRP: C-reactive protein; RT-PCR: real-time reverse transcription-polymerase chain reaction; SARS-CoV-2: severe acute respiratory syndrome coronavirus 2; AST: aspartate aminotransferase; ALT: alanine aminotransferase; LDH: lactate dehydrogenase; VDRL: venereal disease research laboratory; ESR: erythrocyte sedimentation rate; ARDS: acute respiratory distress syndrome; SFJDC: Shufeng Jiedu Capsule; IV IG: intravenous immunoglobulin; HIV: human immunodeficiency virus; IU/L: international units per liter; pg/mL: picogram per milliliter; mmol/L: millimoles per liter.

Study	Demographics	Clinical presentation	Imaging studies	Lab findings	Treatment	Outcome
Park et al. [12]	10-year-old Korean female	Mild pneumonia. Fever, cough with a small amount of sputum	CXR: no infiltrations were noted on the initial and three follow-up CXR. Chest CT: patchy or nodular consolidations with peripheral ground-glass opacities in subpleural areas of right lower lobe	WBC 4,080/μL (37.3% lymphocytes), CRP < 0.40 mg/dL. RT-PCR for SARS-CoV-2 positive	Isolated in a negative-pressure room	Good prognosis with no complications. The patient was cured and alive
Zhang et al. [25]	75-year-old Chinese male	Severe COVID-19 pneumonia with ARDS. Hypothermic and bradycardia	Chest CT: white “septal line” that shows cellulosic exudation in the surface of lung lobes	-	Non-invasive ventilation with oxygenation index of 100 mmHg	Complications: ARDS, septic shock, and multiple organ dysfunction syndrome. The patient was cured and alive
Cheng et al. [20]	55-year-old Taiwanese male	Mild pneumonia with sore throat, dry cough, fatigue, and low-grade fever	CXR: b/l perihilar infiltration and ill-defined patchy opacities. Chest CT: tenacious COVID-19 pneumonia	RT-PCR for SARS-CoV-2 positive. Lymphopenia (531 cells/µL), elevated AST (78 IU/L), ALT (41 IU/L), CRP (7.8 mg/L), and LDH (295 IU/L)	Supportive: oxygen supplement, saline infusion. Ceftriaxone 2 g loading dose and 2 g IV daily, replaced on day 17 by amoxicillin/clavulanate 875/125 mg BID for one week	Good prognosis with no complications. The patient was cured and alive
Wei et al. [26]	40-year-old Korean female	Mild pneumonia with fever, chest tightness, fatigue, and cough	CXR: normal in both lungs. Chest CT: ground-glass opacities in the subpleural area of the right lower lobe and the left lung was normal	Normal WBC (4170/µL), neutrophils (59.6%), and lymphocytes (30.9%). Elevated hematocrit (0.456), elevated glucose (7.3 mmol/L), and elevated CRP (8.00 mg/L). Influenza A antigen, and RT-PCR for SARS-CoV-2 positive	Isolated in a negative-pressure room. Lopinavir 200 mg/capsule, two capsules BID. Piperacillin + tazobactam	Good prognosis with no complications. The patient was cured and alive
Silverstein et al. [21]	56-year-old male	Mild pneumonia with fever, non-productive cough, mild hemoptysis, and significant rhinorrhea	CXR: patchy b/l, peribronchovascular, ill-defined opacities in all lung zones	Mild thrombocytopenia, normal hemoglobin concentration, and normal WBC. ALT 29 IU/L and lactate concentration 1·1 mmol/L. Influenza virus A and influenza virus B, parainfluenza virus, respiratory syncytial virus, adenovirus, and human metapneumovirus: negative. RT-PCR for SARS-CoV-2 positive	Isolated in a negative-pressure room	Good prognosis with no complications. The patient was cured and alive
Lim et al. [23]	54-year-old Korean male	Mild pneumonia with chills, muscle pain, fever, and dry cough	CXR: no haziness. Chest CT: Small consolidation in the right upper lobe and ground-glass opacities in both lower lobes	Leptospira, Hantaan virus, tsutsugamushi, malaria, Mycobacterium tuberculosis, HIV, and VDRL test: negative. RT-PCR for SARS-CoV-2 positive	Isolated in a negative-pressure room. Lopinavir 200 mg/ritonavir 50 mg, two tablets per oral	Good prognosis with no complications. The patient was cured and alive
Duan and Qin [27]	46-year-old Chinese female	Mild pneumonia with fever without chills and rigor, nasal discharge, cough, and myalgia	Chest CT: multiple b/l and peripheral ground-glass opacities in the superior segments of both lower lobes without sparing of subpleural regions	Normal WBC (52.9% neutrophils, 28.3% lymphocytes). Elevated blood levels for CRP (6.4 mg/L), ESR (27 mm/h), and D-dimer (566 ng/mL). RT-PCR for SARS-CoV-2 positive	Isolated in a negative-pressure room. Interferon inhalation	Good prognosis with no complications. The patient was cured and alive
Wang et al. [22]	32-year-old Chinese male	Mild pneumonia with fever, fatigue. dizziness, and constipation	Chest CT: ground-glass opacities and consolidation, b/l pneumonia	Normal WBC 4,230/μL (30.3% lymphocytes, 57.2% neutrophils). RT-PCR for SARS-CoV-2 positive	Oxygen therapy and antibiotics. Lopinavir/ritonavir/arbidol/SFJDC	Good prognosis with no complications. The patient was cured and alive
	19-year-old Chinese male	Mild pneumonia with fever, fatigue, cough, nasal congestion, rhinorrhea	Chest CT: ground-glass opacities and consolidation, unilateral pneumonia	Normal WBC 6,480/μL (30.6% lymphocytes, 57% neutrophils). RT-PCR for SARS-CoV-2 positive	Oxygen therapy and antibiotics. Lopinavir/ritonavir/arbidol/SFJDC	Good prognosis with no complications. The patient was cured and alive
	63-year-old Chinese male	Severe pneumonia with fever and cough	Chest CT: ground-glass opacities and consolidation, unilateral pneumonia	Normal WBC 4.400/μL (24.5% lymphocytes, 50% neutrophils). RT-PCR for SARS-CoV-2 positive	Oxygen therapy and antibiotics. Lopinavir/ritonavir/arbidol/SFJDC	Good prognosis with no complications. The patient was cured and alive
	63-year-old Chinese female	Severe pneumonia with fever, cough, dizziness, and constipation	Chest CT: ground-glass opacities and consolidation, b/l pneumonia	Normal WBC 6,840/μL (low lymphocytes 6.1% ,high neutrophils 93%). RT-PCR for SARS-CoV-2 positive	Oxygen therapy mechanical ventilation. Antibiotic treatment. Lopinavir/ritonavir/arbidol/SFJDC. IV IG therapy	Complication: hypoxia. The patient was cured and alive
Shi et al. [33]	42-year-old Chinese male	Mild pneumonia with high-grade fever (39.6°C), cough, and fatigue	CXR: opacities in the left lower and right upper lobes. Chest CT: multifocal b/l ground-glass opacities	Low WBC 2,880/μL (56.6% neutrophils, 32.1% lymphocytes, and 10.2% monocytes). Elevated CRP 158.95 mg/L, ESR (38 mm/h), and serum amyloid A protein (607.1 mg/L). Elevated AST (53 IU/L), ALT (60 IU/L). RT-PCR for SARS-CoV-2 positive	Supportive care. Antivirals: ganciclovir, oseltamivir. Antibiotics: meropenem, linezolid	Good prognosis with no complications. The patient was cured and alive
Fang et al. [24]	45-year-old Chinese female	Mild pneumonia with fever and cough	Chest CT: multiple b/l areas of peripheral consolidation. There was interlobular septal thickening with a crazy-paving appearance and bronchiectasis. The adjacent pleura was thickened, without mediastinal lymphadenopathy or pleural fluid	Increased neutrophil ratio (81.2%), decreased lymphocyte ratio (12.8%), Increased ESR (24 mm/h), normal D-dimer concentration, and increased lymphokine interleukin 6 (27.47 pg/mL). RT-PCR for SARS-CoV-2 positive	Antiviral and symptomatic treatment	Good prognosis with no complications. The patient was cured and alive
	32-year-old Chinese male	Mild pneumonia with fever and cough	Chest CT: subpleural right lower lobe consolidation. There was bronchiectasis with reactive thickening of the adjacent pleura. There was no mediastinal lymphadenopathy or pleural effusion	Normal complete blood count values. Normal CRP, normal D-dimer concentration. Lymphokine interleukin 6 increased (413.6 pg/mL). RT-PCR for SARS-CoV-2 positive	Antiviral and symptomatic treatment	Good prognosis with no complications. The patient was cured and alive
Chen et al. [10]	39-year-old Chinese male	Severe pneumonia with fever (up to 37.7°C) and aggravated cough with frothy white sputum, poor mental states, and shortness of breath	Chest CT: mild pleural effusion in the left lung, an increase in the density of ground-glass opacities, and an extension of the patchy area	Influenza virus, respiratory syncytial virus, adenovirus, metapneumovirus, Mycoplasma pneumoniae, Chlamydophila pneumoniae, and Legionella: negative. RT-PCR for SARS-CoV-2 positive	Antiviral treatment	Complication: type I respiratory failure. The patient was cured and alive
	21-year-old Chinese female	Mild pneumonia with intermittent febrile cough, chills, fever (up to 40°C), and frothy white sputum	Chest CT: patchy pulmonary opacities below the pleura in b/l lung field	Influenza virus, respiratory syncytial virus, adenovirus, metapneumovirus, Mycoplasma pneumoniae, Chlamydophila pneumoniae, and Legionella: negative. RT-PCR for SARS-CoV-2 positive	Antiviral treatment	Good prognosis with no complications. The patient was cured and alive
Kim et al. [9]	35-year-old Chinese female	Mild pneumonia with fever, chill, and myalgia, nasal congestion, cough, sputum, pleuritic chest discomfort, and watery diarrhea	CXR: no infiltrations. Chest CT: multiple, ground-glass opacities located in both subpleural spaces	Leukopenia, thrombocytopenia, and liver enzyme elevation. RT-PCR for SARS-CoV-2 positive	Lopinavir 400 mg/ritonavir 100 mg	Good prognosis with no complications. The patient was cured and alive
Liu et al. [13]	41-year-old Chinese male	Mild pneumonia with fever	CXR: normal. Chest CT: multifocal ground-glass opacities in the perihilar and subpleural regions of both lungs	Normal WBC, normal liver enzymes. RT-PCR for SARS-CoV-2 positive	Isolated in a negative-pressure room	Good prognosis with no complications. The patient was cured and alive
Holshue et al. [14]	35-year-old Chinese male	Mild pneumonia with cough, subjective fever, and persistent dry cough. Nausea and vomiting, loose bowel movement, and abdominal discomfort	CXR: pneumonia in the lower lobe of the left lung	Influenza A and B, parainfluenza, respiratory syncytial virus, rhinovirus, adenovirus, and four common coronavirus strains (HKU1, NL63, 229E, and OC43): negative. Leukopenia, mild thrombocytopenia, and elevated levels of creatine kinase. Elevated alkaline phosphatase (68 IU/L), ALT (105 IU/L), AST (77 IU/L), and LDH (465 IU/L). RT-PCR for SARS-CoV-2 positive	Isolated in a negative-pressure room supportive care. Vancomycin 1,750 mg loading dose followed by 1 g IV every eight hours. Cefepime IV every eight hours	Good prognosis with no complications. The patient was cured and alive
Lei et al. [34]	33-year-old Chinese female	Mild pneumonia with fever and cough	Chest CT: multiple peripheral ground-glass opacities in b/l lungs that did not spare the subpleural regions	Leucopenia (WBC: 2,910/μL, 70% neutrophils and 0.1% eosinophils). Elevated CRP (16.16 mg/L), ESR (29 mm/h), and D-dimer (580 ng/mL). RT-PCR for SARS-CoV-2 positive	Isolated in a negative-pressure room. Interferon inhalation	Good prognosis with no complications. The patient was cured and alive
Lin et al. [8]	35-year-old Chinese male	Mild pneumonia with fever and cough	Chest CT: multiple regions of patchy consolidation and ground-glass opacities with indistinct border in b/l lungs. The lesions were distributed along the bronchial bundles or within the subpleural lung regions. Neither pleural effusion nor lymphadenopathy was found	Normal WBC (5,520/µL), increased neutrophils (76.2%), decreased lymphocytes (16.1%). Elevated glucose (7.4 mmol/L), elevated CRP (14.00 mg/L). Respiratory syncytial virus, adenovirus, influenza A virus, Mycoplasma pneumoniae, Chlamydia pneumoniae, Legionella pneumophila, parainfluenza virus, influenza A and B virus antigen: negative. RT-PCR for SARS-CoV-2 positive	Isolated in a negative-pressure room	Good prognosis with no complications. The patient was cured and alive
	39-year-old Chinese male	Mild pneumonia with fever and throat discomfort	Chest CT: small ill-defined ground-glass opacities in b/l lower lung lobes. The lesion in the left lower lobe was located in the subpleural region while the one in the right lower lobe was distributed along the bundles. Neither pleural effusion nor lymphadenopathy was found	Normal WBC (5,320/µL), normal neutrophils (67.6%), normal lymphocytes (24.5%). Decreased AST (14 IU/L), elevated glucose (6.8 mmol/L), and normal CRP (4.00 mg/L). RT-PCR for SARS-CoV-2 positive	Isolated in a negative-pressure room	Good prognosis with no complications. The patient was cured and alive

## Conclusions

The results of our systematic review of published case reports of pneumonia in 2019-nCoV positive patients will help healthcare professionals to understand the pathophysiology and transmission, clinical presentation, imaging and laboratory findings, and treatment. Most patients presented with fever, fatigue, and dry cough; some present with nasal congestion, runny nose, myalgia, and chills. The most common finding on chest CT was ground-glass opacities, involving bilateral lungs with the peripheral distribution. Lymphopenia was the most common lab finding, and along with other inflammatory markers in patients with greater illness severity. The use of LPV/RTV and remdesivir was associated with significant clinical improvement in severe pneumonia. Nonetheless, we need more RCTs and treatment guidelines for developing effective management of the 2019-nCoV and improve patient outcomes by reducing mortality in high-risk patients.
